# Bridging the gap between child mental health need and professional service utilisation: Examining the influence of mothers’ parental attributions on professional help-seeking intentions

**DOI:** 10.1007/s00787-020-01682-6

**Published:** 2020-11-19

**Authors:** Vilas Sawrikar, Antonio Mendoza Diaz, Lucy Tully, David J. Hawes, Caroline Moul, Mark R. Dadds

**Affiliations:** 1 School of Psychology, Faculty of Science, University of Sydney, Sydney, Australia; 2 School of Psychiatry, Faculty of Medicine, University of New South Wales, Sydney, Australia; 3 Department of Clinical Psychology, School of Health in Social Science, University of Edinburgh, Edinburgh, United Kingdom

**Keywords:** Child mental health, Help-seeking, Treatment engagement, Treatment adherence, Parental attributions

## Abstract

There is a significant gap between the need for child mental health services and use of these services by families. Parental attributions may play a role in this. This study examined whether mothers’ attributions about their child’s problems influence professional help-seeking intentions in a general sample of community mothers. Secondary analysis re-examined this hypothesis in a subgroup of mothers of children with clinically elevated mental health symptoms. Cross-sectional survey data were collected from mothers (*N* = 184) of children aged between 2 and 12 years recruited from the community. Mothers completed self-report questionnaires measuring parental attributions: child-responsible attributions and parental self-efficacy; professional help-seeking intentions; and psychosocial covariates: child mental health, mothers’ anxiety and depression, child age, gender, marital status, education, and professional help-seeking experience. Hierarchical regression modelling indicated that parental attributions explained professional help-seeking intentions after controlling for covariates in both the general sample (Δ*F* = 6.07; *p* = .003) and subgroup analysis (Δ*F* = 10.22, *p* = .000). Professional help-seeking intentions were positively associated with child-responsible attributions (β = .19, *p* = .002) but not parental self-efficacy (β =  – .01, *p* = .865) in the general sample, while positively associated with child-responsible attributions (β = .20, *p* = .009) and negatively associated with parental self-efficacy (β =  – .16, *p* = .034) in the subgroup analysis. Findings were independent of the presence of clinically elevated symptoms, problem type, and severity. Overall, the findings support models suggesting that parental attributions have a role in professional help-seeking for child mental health problems.

**Electronic supplementary material:**

The online version of this article (10.1007/s00787-020-01682-6) contains supplementary material, which is available to authorized users.

## Introduction

Mental health problems are prevalent among children, with research indicating that approximately one in seven children will meet criteria for a mental health disorder [1]. These figures are likely to underestimate mental health concern among children as they do not include those with subclinical symptoms warranting treatment [2]. Further, with around half of all lifetime mental disorders emerging in childhood

before 12 years of age, intervening in these early years is critical to preventing a life course of poor mental health [3]. However, despite high rates of mental health need during childhood, parents appear to be reluctant to engage health professionals for help [4]. This has prompted calls for strategies to improve parents’ willingness to engage professional help to better meet the needs of children with mental health problems [3, 5]. In line with this, the current study evaluated the influence of parental attributions about the causes of child problems on intentions to engage with professional help, to evaluate whether it may represent a putative target for improving parental engagement with child mental health professional services.

Research indicates that parents are generally unwilling to seek professional help for child mental health problems with parents preferring to manage their child’s problem on their own or seek out informal advice from family and friends [4]. This research suggests parents are more likely to engage in informal help that is not informed by evidence-based guidelines than to receive professional help [5, 6]. If parents do seek professional help, they are also unlikely to sufficiently adhere to and engage with treatment for full implementation of evidence-supported treatments [5]. Therefore, reviews of mental health service utilisation have, emphasised that, to reduce the discrepancy between need and service use, it is important to investigate factors influencing parents’ motivation and willingness to seek and engage with professional help [7].

Help-seeking models relevant for child mental health suggest that child symptoms alone are not enough to motivate service utilisation. These models hypothesise that parents’ motivation to seek help for child problems result from recognising and appraising child problems as problematic, including the causes (attributions), meaning (worry about child behaviour), and perceptions of child problems (e.g., perceived severity, susceptibility), which subsequently lead to decisions to seek help [7–10]. Further, these models hypothesise that parents’ progress from decisions to seek help to actual service utilisation by evaluating structural barriers and facilitators, including availability of professional help, economic factors, and service characteristics such as waiting times and referral care pathways [e.g., primary care to specialist services; 8, 10]. In recent years, researchers have moved to consider other illness representations (consequences, identity, emotional impact) alongside parental attributions (internality, stability, controllability) as ‘cognitive representations of illness’ in understanding parents’ engagement of child mental health treatment [11, 12]. These models emphasise the need to understand parents’ experience of and attributions about their own child’s problems in parents’ adherence to treatment.

In somewhat parallel lines of research to the study of parental help-seeking, social cognitive theories of family health behaviour have also linked parental attributions to parents’ motivation to engage in child mental health treatment. Specific attention is given to two types of parental attributions in predicting individual differences in parent’s motivation to engage with treatment: ‘child-responsible’ and ‘parent-referent’ attributions [9, 13]. This was based on a review of parental attributions indicating that parents of children with behavioural and emotional problems are more likely to attribute the cause of their child problems to intentional, stable, and dispositional child factors (‘child-responsible attributions’) and report lower sense of competence in managing child behaviour (‘parent-referent attributions’) compared to parents of non-problematic children [13]. Based on their review, Morrissey-Kane and Prinz [13] tentatively proposed that parents would be less willing to utilise child mental health treatment if they view the child as blameworthy/responsible for problematic behaviour while also reporting lower competence in managing behaviour, as these attributions would lead parents to feel pessimistic about their child improving and/or that they were ineffective change agents in improving child behaviours.

The study of parental attributions and help-seeking for professional help in the context of child mental health is notably lacking to date [12]. Furthermore, there is significant heterogeneity in the methods used across studies. We note that in one line of research, parental attributions were examined in relation to whether they are associated with parents’ actual utilisation of child mental health services or other forms of professional help [14–18]. However, research examining actual service utilisation is limited in examining processes related to understanding parents’ willingness to seek professional help occurring prior to referral to service [19].

Researchers subsequently shifted their attention to examining parents’ intentions to seek professional help (‘professional help-seeking intentions’) as a preceding factor to actual service utilisation [e.g., [20]]. Using community samples of parents, studies examined parents’ intentions to seek professional help for hypothetical child behaviour and emotional problems presented in vignettes. Child-responsible attributions were measured by parents’ explanations for problems presented in the vignettes. Parent-referent attributions were assessed using self-report measures of parental self-efficacy. In partial support for parental attributions to influence professional help-seeking intentions, Maniadaki et al. [21] found that perceived severity of child problems, measured using an aggregate scale of child-responsible attributions, perceived severity, and parental concern, was associated with professional help-seeking intentions. The specific influence of child-responsible attributions was unclear, but results suggest further research is warranted. On the contrary, studies which measured child-responsible attributions based on parental endorsement of specific child causes (e.g., personality, acquired habits, skills deficits, or intention to irritate parents) did not find evidence of a relationship between child causal factors and help-seeking intentions [22, 23]. Parental self-efficacy was also not associated with parents’ help-seeking intentions, at least for description of symptoms of Attention Deficit Hyperactivity Disorder [21, 24]. Taken together, there is mixed findings about whether parental attributions influence parents’ intentions to seek professional help.

On the basis of the mixed findings and paucity of research to date, more research is needed, especially in relation to personal circumstantial factors that may influence the relationship between parental attributions and professional help-seeking intentions [15]. For instance, previous measurement of child-responsible attributions relied on hypothetical vignettes (‘stimulus-dependant attributions’), thus the influence of parental attributions reflecting parents’ own experiences (‘memory-dependent attributions’) were not evaluated [25]. Raviv and colleagues (2003) demonstrated that mothers can have different intentions to seek professional help when assessment questions refer to their own or another person’s child. Likewise, the experience of parenting a child with mental health symptoms was not considered, including problem type and severity levels, which also predict help-seeking intentions and behaviour [9, 15, 22]. Finally, previous research focused on intentions to seek professional help only, while excluding other dimensions of treatment engagement such as adherence [5].

To address these limitations, the current study tested whether parental attributions contributed to explaining individual differences in parents’ intentions to engage with, and adhere to, professional help (i.e. professional help-seeking intentions) using data collected from mothers in the community. Both child-responsible and parent-referent attributions were assessed using measures of memory-dependent attributions. Our primary hypothesis was that mothers’ parental attributions was significantly associated with professional help-seeking intentions after controlling for psychosocial covariates, child mental health, mothers’ emotional health, child age, gender, marital status, education, and professional help-seeking experience, known to influence help-seeking behaviours (Hypothesis 1). Specifically, greater help-seeking intentions were expected to be associated with a positive parental attribution style, namely lower child-responsible attributions and higher parental self-efficacy [13]. We also tested whether the association between parental attributions and professional help-seeking intentions was dependent on reports of clinically elevated child mental health symptoms (Hypothesis 2). A second analysis repeated the test of whether parental attributions were associated with professional help-seeking intentions for those families with evidence of child mental health need using a subgroup of mothers reporting on a child with clinical elevated mental health symptoms (Hypothesis 3). This time the subgroup analysis tested whether problem type and severity moderated the relationship between parental attributions and help-seeking intentions in order to disentangle child factors which may influence the relationship between parental attributions and professional help-seeking intentions (Hypothesis 4).

## Method

### Study design and procedures

The data for this study were drawn from the Sydney Parenting Experience Survey, a cross-sectional community survey conducted in Sydney, Australia. The Sydney Parenting Experience Survey was developed as a pilot survey to assess parenting and child mental health literacy in the community. Eligibility criteria were (i) parent of a child between the ages of 2 and 16 years, (ii) living in Sydney, and (iii) competency in English. Data were collected in accordance with ethical guidelines provided by the Human Research Ethics Committee. Survey responses were pseudo-anonymised and stored confidentially in a password-protected, secured university network only accessible to researchers involved in the study. Participation in the study was voluntary, and informed consent was obtained prior to the start of the survey. Parents were informed to complete the survey at their convenience, including the option to take breaks and return later to complete the survey. Time data collected through Qualtrics (see recruitment section below) indicated that the median time to complete the survey was 24 min (95%CI 23–27 min).

### Recruitment

**Fig. 1:**
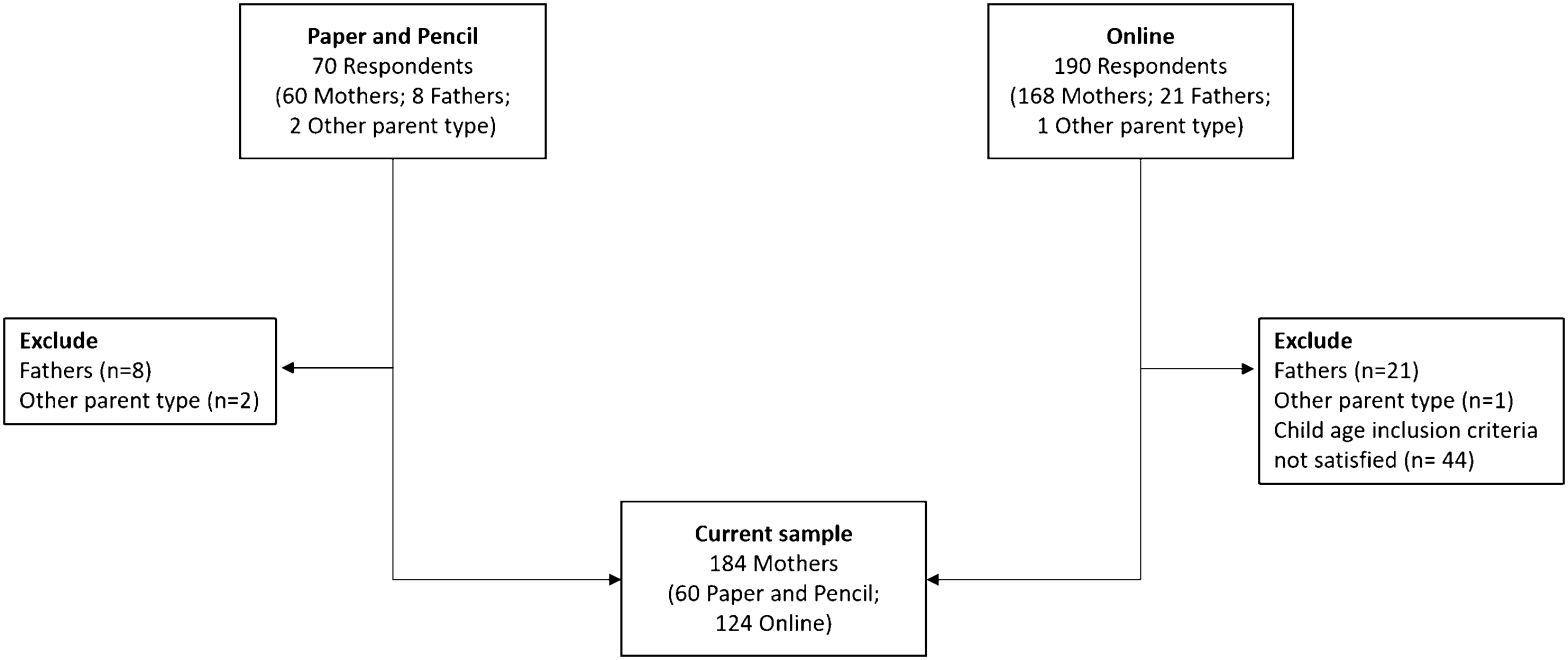
Participant flow chart for the current study

Participants were recruited between 2015 and 2017 in a series of recruitment rounds using convenient nonprobability sampling [26]. Two methods of community contact were used (Fig. 1). The first method collected responses from parents via paper-based surveys distributed through teachers in child day care centres (*n* = 70 respondents) located within proximity of the research site. Day care centres were all in metropolitan urban areas ranging from the 6th to 10th decile of national socio-economic advantage according to Socio-economic Indexes for Areas [27]. Responses were subsequently entered in an electronic database. The second method consisted of questionnaire responses collected at community parenting workshops held at the research site at no cost (*n* = 190 respondents). Advertising for the parenting workshops included university email distribution chains and public event notice boards and registration to attend the workshop was managed by a third-party website (Eventbrite). Responses from parents attending the workshops were collected electronically via the Qualtrics online survey platform via a link advertised at the end of the workshops. Response rates could not be calculated as information regarding the number of parents approached in day care centres and who viewed the survey link at the workshops was not recorded.

### Participants in current study

Participants included in this study were mothers reporting on a child between 2 and 12 years of age (*N* = 184; paper and pencil:* n* = 60; online survey: *n* = 124). Table 1 summarises the child, parent, and family characteristics of participants in the current study. Slightly more boys than girls were included in the sample (boys = 56%) and most of the children in the study had one sibling (no siblings: 28.3%; 1 sibling: 48.9%; 2 siblings: 20.7%; 3 siblings: 2.2%). Almost all mothers reported being married or in a defacto relationship (92.9%) and most mothers had university-level education (82.1%) working as ‘Professionals’ (63.0%). Finally, most mothers indicated that English was the primary language spoken at home (81.5%), followed by an Anglo-European language (12.0%), Asian/South Asian language (3.8%), and combined English and Anglo-European language (2.7%). Of the sample, 115 mothers (62.5%) reported on a child with clinically elevated mental health symptoms determined by mothers’ SDQ scores within the borderline or abnormal range for at least one SDQ problem subscale [symptoms present: 1 = yes; 0 = no; 15]. Mothers of children with elevated mental health symptoms on at least one scale on the SDQ (clinically elevated symptoms present = yes) were used for the subgroup analysis in this study.

We note that fathers were not included in the current analysis due to inadequate power to conduct reliable hypothesis testing. Low father participation reflected the challenges of recruiting fathers. While both mothers and fathers were requested to participate, recruitment through pre-schools and community workshops were held during the day where mothers were likely to attend and fulfil participation requirements.

**Table 1:** Child, parent, and sociodemographic data collected from mothers in the community sample

Sociodemographic variables	
Child’s age *M* (SD)	4.67 (2.56)
Child’s gender *N* (%)	
Boys	104 (56.5%)
Girls	80 (43.5%)
Child mental health symptoms	
Clinical elevated*	115 (62.5%)
Normal	69 (37.5%)
Mother’s anxiety/depression M (SD)	3.43 (2.9)
Mother’s education N (%)	
Secondary	8 (4.3%)
College/Tafe	25 (13.6%)
University	151 (82.1%)
Mother’s occupation N (%)	
Managers	21 (11.4%)
Professionals	116 (63.0%)
Technicians and trades workers	1 (.5%)
Community and personal service workers	3 (1.6%)
Clerical and administrative workers	16 (8.7%)
Sales workers	3 (1.6%)
Other	24 (13.0%)
Marital status *N* (%)	
Married/defacto	171 (92.9%)
Single	13 (7.1%)
Number of siblings	0.97 (0.76)

*M* mean, *SD *standard deviation

*Presence of clinically elevated child mental health symptoms was determined by mothers’ SDQ scores within the borderline or abnormal range for at least one SDQ problem subscale (conduct problems, emotional problems, hyperactivity, peer problems). ^*ǂ*^Mothers occupation was classified according to Australian and New Zealand Standard Classification of Occupations [58]. Other occupations included self-employed (7 [29%]), student (8 [33%]); stay-at-home mother (6 [25%]), and not classifiable (3 [13%])

## Measures

**Demographic information.** Information related to mother’s education level, marital status, number of siblings, child gender, and child age was collected as part of the survey. Marital status was recoded into single or two parent status where participants were a single parent if they reported to be separated, divorced, or single.

**Child mental health**. Child mental health was measured using the four mental health problem subscales of the Strengths and Difficulties Questionnaire (SDQ): Conduct Problem scale (oppositional, defiant, and rule-breaking behaviours), Emotion Problem scale (somatic and anxiety symptoms), Hyperactivity (distractibility and hyperactivity), and Peer Problems scale (peer relationships) [SDQ; [28]]. Each scale consists of five items scored on a 3-point scale from 0 (‘not true’) to 2 (‘certainly true’). Research has shown that the SDQ subscales are well validated in international and Australian family populations [29, 30]. Each of the subscales had moderate to good internal consistency in this study: Conduct Problems (α = 0.64), Emotional Problems (α = 0.75), Hyperactivity (α = 0.77), and Peer Problems (α = 0.64). Individual SDQ scores were subsequently coded by severity range [normal, borderline, abnormal; 30], and those in the borderline/abnormal range were included in the ‘clinically elevated’ range.

**Mother’s emotional health**. A single self-report analogue item was developed for this study to assess mothers’ emotional health along dimensions of anxiety and depression. The decision to use a single item was based on mother’s mental health not being a primary interest area of the survey and to reduce responder burden in a community-based study. Participants rated the extent to which anxiety/depression had affected their life, scored on an 11-point scale from 0 (‘none at all’) to 10 (‘completely’) (“To what extent has the life of the Mother been affected by depression and anxiety”). Mothers in the current sample indicated that anxiety and depression had a low impact in their life (distribution of responses: 0–3: 58.2%, 4–7: 30.4%; 8–10: 11.4%).

**Parental self-efficacy**. The original Parental Locus of Control (PLOC) measure is a 47-item questionnaire assessing self-efficacy in parent–child interactions across five dimensions of Parental Efficacy, Parental Responsibility, Child Control of Parents’ Life, and Parental Belief in Fate/Chance [31]. A short-form version (PLOC-SF) was developed for the current study to help reduce responder burden where ten items were chosen based on the original study by selecting two items from each of the five dimensions that had the highest loadings on that dimension. Participants were required to endorse items on a 5-point scale ranging from 1 ('strongly agree') to 5 ('strongly disagree'). A total score was calculated by summing responses across the ten items with higher scores indicating an internal locus of control similar to other brief measures of PLOC [32]. A similar method of developing a 30-item short-form version of the PLOC [31] was reported by Lovejoy et al. [33], and their short-form version was shown to have good internal consistency and test–retest reliability. An evaluation of the current 10-item PLOC-SF measure using the current sample indicated that it had acceptable internal consistency (α = 0.63), high test–retest reliability (*r* = 0.74),^1^ and good convergent validity from correlational analysis showing significant negative associations with child-responsibility attributions and SDQ scales of conduct problems, emotional symptoms, hyperactivity, and peer problems as expected (see supplementary Table 1).

**Child-responsible attributions**. Child-responsible attributions for child problems were measured using the Parent Attribution Measure (PAM), a 12-item self-report measure designed to assess intentionality/controllable, permanence, and disposition attributions for child problem behaviour in the context of their own parenting experiences [34]. Participants rated their agreement with statements on a 3-point scale that ranged from 1 (‘not at all true’) to 3 (‘certainly true’). Positively worded items were reverse scored and all items were used to calculate a higher-order Total Scale of child-responsible attributions for problem behaviours (e.g., under the child’s control, permanent, and dispositional) similar to other self-report measures of parental attributions [35]. The PAM previously showed excellent reliability in clinical samples involving parents of children with behavioural problems [36, 37]. The internal consistency for the total scale in the current community sample was acceptable (α = 0.66).

**Professional help-seeking intentions**. Mothers’ professional help-seeking intentions were measured using an adapted version of the general help-seeking questionnaire [GHSQ; [38]]. The approach used to adapt the GHSQ was adopted from methods described in Rickwood, Deane, Wilson, & Ciarrochi [39] which combined items assessing the quantity and quality of previous counselling experience with items assessing future help-seeking intentions. The aim of adapting the GHSQ for the current study was to focus on intentions to seek and engage professional help only rather than help from multiple nonprofessional sources. The specific steps were as follows: (1) the matrix-style Likert scale assessing future likelihood of seeking help from multiple sources (‘how likely is it that you would seek help from the following people? e.g., intimate partner, friend, parent etc.’) was replaced with three Likert scale items measuring future professional help-seeking intentions (‘In the future: how likely are you to seek professional help; how likely are you to stick with the professional help; how likely are you to follow the recommendations provided to you’); (2) analogue items assessing quantity and quality of previous counselling experience were changed to four Likert scale items asking participants to rate their previous professional help-seeking experience (‘In the past: how likely were you to seek professional help; how likely were you to stick with the professional help; how likely were you to follow the recommendations provided to you; how likely were you to find the professional help worthwhile’); (3) the problem provided in the instructions was changed from ‘if you were having a personal or emotional problem’ to ‘if you or your family experience problems’. Participants rated their agreement to statements on a 5-point scale that ranged from 1 ('extremely unlikely') to 5 ('extremely likely'). The questionnaire stated that information from parenting workshops should not be a part of responses. Internal consistency of scales assessing future professional help-seeking intentions (α = 0.85) and previous professional help-seeking experience (α = 0.87) were high.

### Statistical analysis

Descriptive statistics were calculated using SPSS version 24 to initially examine distribution of scores for severity of child mental health symptoms, professional help-seeking intentions, previous professional help-seeking experiences, and parental attributions in the full sample. Bivariate correlation analyses examined univariate associations among variables. Chi-square tests were used to evaluate whether child sex differences existed in child mental health severity ratings, while one-way ANOVAs tested mean level differences across child gender for professional help-seeking intentions, previous professional help-seeking experiences, and parental attributions.

The hypothesis that parental attributions contributed to explaining mothers’ future professional help-seeking intentions above other known predictors (Hypotheses 1) was first evaluated by hierarchical linear regression modelling using the full sample (SPSS version 24). The adapted GHSQ scale measuring future professional help-seeking intentions was entered as the dependent variable. Covariate predictors entered in the first regression block were single parent status, maternal factors (level of education, anxiety/depression), child demographics (age, gender), previous professional help-seeking experience, and child mental health status (clinically elevated symptoms (borderline/abnormal) = 1, normal = 0) [40]. Child-responsible attributions (PAM Total Scale score) and parent-referent attributions (PLOC-SF Total score) were entered in the second regression block. To test Hypothesis 2, the interaction terms of clinically elevated symptoms present $\times$ PAM Total Scale z-score and clinically elevated symptoms present $\times$ PLOC-SF Total z-score were entered in the third regression block.

For the subgroup of mothers reporting on a child with clinically elevated mental health symptoms (*n* = 115), hierarchical linear regression modelling was again used to examine whether parental attributions contributed to explaining professional help-seeking intentions above other known predictors, including problem type and severity (Hypothesis 3). The first regression block consisted of covariate predictors with child mental health status replaced by SDQ severity ratings (normal, borderline, abnormal) for each problem subscale. It should be noted that since categorising children into the clinically elevated subgroup was based on scores on at least one SDQ problem subscale within the borderline or abnormal range, children could have had scores within the normal range for the other problem subscales. The second regression block consisted of parental attribution variables. To test Hypothesis 4, the potential moderating effect of problem type and severity was examined by adding independent blocks testing the significance of additional interaction term(s) representing the hypothesised moderator effect (e.g., PAM Total Scale z-score $\times$ SDQ conduct severity range; PAM Total Scale z-score $\times$ SDQ emotional severity range; PAM Total Scale z-score $\times$ SDQ hyperactivity severity range; and PAM Total Scale z-score $\times$ SDQ peer problems severity range; steps were repeated for parental self-efficacy).

The main effect of adding parental attributions (block 2) and interaction terms (block 3) was ascertained by the significance of F_Change_ (α = 0.05). Post-hoc Scheffe tests examined the significance of individual parental attribution variables and interaction terms to control for familywise error associated with performing multiple tests simultaneously. The Scheffe procedure provides a conservative post-hoc test minimising risk of false discovery [41]. Assumptions underlying hypothesis testing using linear regression were judged by inspecting the distribution characteristics of the standardised residuals [42]. Across each model where the role of parental attributions was tested, standardised residuals were normally and linearly distributed with no evidence of heteroscedasticity.

## Results

### Descriptive statistics for the full community sample

Descriptive statistics for child mental health symptom severity, future professional help-seeking intentions, previous professional help-seeking experience, and parental attributions are reported in Table 2. Overall for the sample, the mean scores for child mental health symptoms on the SDQ were within the normal range [30]. No gender differences were found within variables. Correlations among variables are provided in Table 1 in supplementary information. Child-responsible attributions and parental self-efficacy were not associated with future professional help-seeing intentions at the univariate level.

**Table 2:** Descriptive statistics for child mental health symptoms, previous professional help-seeking experience, future professional help-seeking intentions, and parental attributions by child gender

Variable	Range	*N* (%) / mean (SD)		Statistics
		Boys	Girls	
Conduct problems	Normal	76 (73.1%)	65 (81.3%)	χ^2^(2) = 2.41
	Borderline	11 (10.6%)	8 (10%)	
	Abnormal	17 (16.3%)	7 (8.8%)	
Emotion problems	Normal	68 (65.4%)	53 (66.3%)	χ^2^(2) = .09
	Borderline	7 (6.7%)	6 (7.5%)	
	Abnormal	29 (27.9%)	21 (26.3%)	
Hyperactivity	Normal	71 (68.3%)	57 (71.3%)	χ^2^(2) = 2.74
	Borderline	14 (13.5%)	5 (6.3%)	
	Abnormal	19 (18.3%)	18 (22.5%)	
Peer problems	Normal	83 (79.8%)	58 (72.5%)	χ^2^(2) = 3.24
	Borderline	12 (11.5%)	8 (10%)	
	Abnormal	9 (8.7%)	14 (17.5%)	
Previous professional help-seeking experience		15.11 (3.74)	14.21 (4.63)	*F *(1,182) = 2.10
Future professional help-seeking intentions		11.94 (2.71)	12.2 (2.72)	*F *(1,182) = .41
Child-responsible attributions		3.57 (2.85)	3.25 (2.48)	*F *(1,182) = .63
Parental self-efficacy		33.77 (5.46)	33.61 (4.58)	*F *(1,182) = .04

*SD *standard deviation

Mental health symptom ratings measured using the SDQ [30]

*p value < .05

### Full sample analysis of whether mothers’ parental attributions influence future professional help-seeking intentions

Table 3 summarises the results of hierarchical linear regression model using the full sample examining whether mothers’ parental attributions contribute to explaining future professional help-seeking intentions (Hypothesis 1). The addition of variables measuring child-responsible attributions and parental self-efficacy significantly added to explaining mothers’ future help-seeking intentions after controlling for model covariates (Δ*F*(2,174) = 6.07; *p* = 0.003; see Table 3 notes for details of covariates). Post-hoc tests using Scheffe criterion (${F}_{.\mathrm{05,2},174}^{c}$ **=** 6.095) indicated that future professional help-seeking intentions were positively associated with child-responsible attributions (β = 0.19, *F*(1,174) = 9.72) whereby mothers had greater intentions to engage with professional help if they attributed problems to child causal factors (e.g., under the child’s control, permanent, dispositional). Future professional help-seeking intentions were not associated with parental self-efficacy (β =  – 0.01, *F*(1,174) = 0.029).

**Table 3:** Results from full sample analysis of hierarchical regression modelling examining whether mothers’ parental attributions contribute to explaining future professional help-seeking intentions

Variables	B	*t*	Δdf	ΔR^2^	ΔF
*Block 1 (covariates)*			7	.52	26.81*
*Block 2*			*2*	.03	6.07*
Child-responsible attributions	.19**	3.12			
Parental self-efficacy	– .01	– .17			
*Block 3*			2	.02	3.13*
Child-responsible attributions × Child problem present	– .12	– .82			
Parental self-efficacy × Child problem present	– .23***	-2.49			

*n* = 184; dependent variable: future professional help-seeking intentions; covariates: presence of clinically elevated child mental health symptoms (1 = yes; 0 = no), previous professional help-seeking experience, single parent status, education level, depression/anxiety, child age, child gender; B, standardised coefficient; Δ, change; df, degrees of freedom; *p value < .05; **significant Scheffe test result (*t*^2^ > F_c_) where Scheffe = ${F}_{.\mathrm{05,2},174}^{c}$2 × 3.047 = 6.095; ***significant Scheffe test result (*t*^2^ > F_c_) where Scheffe = ${F}_{.\mathrm{05,2},172}^{c}$2 × 3.048 = 6.097

To test Hypothesis 2, moderation analysis tested whether the relationship between parental attributions and future professional help-seeking intentions was moderated by child mental health status (clinically elevated symptoms present: yes/ no). The addition of interaction terms indicated a moderating effect by child mental health status (Δ*F*(2,172) = 3.13; *p* = 0.046). Post-hoc tests using Scheffe criterion (${F}_{.\mathrm{05,2},172}^{c}$ **=** 6.098) indicated that the presence of clinically elevated symptoms moderated the association between parental self-efficacy and future professional help-seeking intentions (β =  – 0.23, *F*(1,172) = 6.18; Table 3 and Figure S1 in online supplementary information). Higher parental self-efficacy appeared to be associated with higher future professional help-seeking intentions for mothers of children with no clinically elevated symptoms. Lower parental self-efficacy appeared to be associated with higher future professional help-seeking intentions for mothers of children with clinically elevated symptoms. Child mental health status did not moderate the relationship between child-responsible attributions and future professional help-seeking intentions (β =  – 0.12, *F*(1,172) = 0.68).

### Subgroup analysis of whether mothers’ parental attributions influence professional help-seeking intentions

The first objective of the subgroup analysis was to repeat the test of whether mothers’ parental attributions contributed to explaining professional help-seeking intentions in a sample of children with clinically elevated mental health symptoms (Hypothesis 3; see results summarised in Table 4). This second analysis was also different to the first by additionally controlling for child mental health problem type and severity. The addition of variables measuring child-responsible attributions and parental self-efficacy significantly added to explaining mothers’ future help-seeking intentions after controlling for model covariates (Δ*F*(2,102) = 10.22; *p* = 0.000; see Table 4 notes for details of covariates). Post-hoc tests using Scheffe criterion (${F}_{.\mathrm{05,2},102}^{c}$ **=** 6.17) indicated that future professional help-seeking intentions were positively associated with child-responsible attributions, as previously described (β = 0.20, *F*(1,102) = 7.00). Parental self-efficacy was significantly negatively associated with future professional help-seeking intention at the less stringent α = 0.05 (β = -0.16, *F*(1,102) = 4.64, *p* = 0.034) whereby mothers with lower self-efficacy had higher future professional help-seeking intentions (Scheffe’s critical level of significance was not satisfied).

**Table 4:** Results from subgroup analysis (mothers of children with clinically elevated mental health symptoms; *n *= 115) of hierarchical regression modelling examining whether mothers’ parental attributions contribute to explaining future professional help-seeking intentions and the moderating influence of child problem type and severity

Variables	B	*t*	Δdf	ΔR^2^	ΔF
*Block 1 (covariates)*			10	.57	13.67*
*Block 2*			*2*	.07	10.22*
Child-responsible attributions	0.20**	2.65			
Parental self-efficacy	–0 .16*	– 2.15			
*Block 3*^*ǂ*^*: Child-responsible attributions* × *child problem type and severity range*			4	.02	1.35
Child-responsible attributions × Conduct problems severity range	– 0.12	– 0.70			
Child-responsible attributions × Emotional problems severity range	– 0.18	– 1.26			
Child-responsible attributions × Hyperactivity severity range	– .06	– 0.37			
Child-responsible attributions × Peer problems severity range	0.31	1.97			
*Block 3*^*ǂ*^*: Parental self-efficacy* × *child problem type and severity range*			4	.01	.50
Parental self-efficacy × Conduct problems severity range	− 0.06	− 0.32			
Parental self-efficacy × Emotional problems severity range	– 0.08	– .49			
Parental self-efficacy × Hyperactivity severity range	– 0 .16	– 1.00			
Parental self-efficacy × Peer problems severity range	.16	1.05			

*Note: n* = 115; independent variable: future professional help-seeking intentions; covariates: child mental health symptoms (SDQ problem type [conduct problems, emotional problems, hyperactivity, peer problems] by severity range [normal, borderline, abnormal]), previous professional help-seeking experience, single parent status, education level, depression/anxiety, child age, child gender; B, standardised coefficient; Δ, change; df, degrees of freedom; *p value < .05; **significant Scheffe test result (*t*^2^ > F^c^) where Scheffe = ${F}_{.\mathrm{05,2},102}^{c}$2 × 3.085 = 6.17^*ǂ*^; interaction terms representing moderator effect were independently tested by adding respective block 3 variables in addition to blocks 1 and 2

The final objective of the subgroup analysis was to examine whether child mental health problem type and severity moderated the relationship between parental attributions and future professional help-seeking intentions (Hypothesis 4). The addition of interaction terms representing the individual moderation effects did not contribute to explaining mothers’ future professional help-seeking intentions after controlling for the main effects of model covariates and mothers’ parental attributions (Child-responsible attributions × child problem type and severity range: Δ*F*(4,98) = 1.35; *p* = 0.256; Parental self-efficacy × child problem type and severity range: Δ*F*(4,98) = 0.50; *p* = 0.737; see Table 4 notes for details of covariates).

## Discussion

The present study sought to extend research examining parental help-seeking by evaluating whether mothers’ parental attributions regarding their own child’s mental health (2–12 years of age) contributed to explaining individual differences in professional help-seeking intentions in a community sample. Table 5 summarises the results. Overall, mothers’ parental attributions explained individual differences in professional help-seeking intentions in both a general sample of community mothers (Hypothesis 1) and a subgroup of community mothers reporting on a child with clinically elevated mental health symptoms (Hypothesis 3). However, different patterns of findings were observed for the two attribution variables used in the study. For child-responsible attributions, mothers had greater future professional help-seeking intentions when they attributed child problems to intentional, permanent, and dispositional factors (child responsible attributions). This was found across both sample groups and was not dependent on the presence of clinically elevated child mental health symptoms (Hypothesis 2) or child problem type and severity (Hypothesis 4). For parental self-efficacy, there was no evidence it was significantly associated with professional help-seeking intentions in the general sample (Hypothesis 1), while a negative association was found in the subgroup analysis whereby lower parental self-efficacy was associated with higher professional help-seeking intentions (Hypothesis 3). The latter finding was not dependent on child problem type and severity (Hypothesis 4).

**Table 5:** Summary of results from hypothesis testing evaluating whether mothers’ parental attributions contribute to explaining professional help-seeking intentions

Hypothesis number	Sample	Specific hypotheses	Result
1	General sample of community mothers	Omnibus test of whether mothers’ parental attributions was associated with professional help-seeking intentions. If supported, ↑professional help-seeking intentions was associated with ↓child-responsible attributions and ↑parental self-efficacy	Omnibus test supported Results contrary to hypothesis: ↑ professional help-seeking intentions associated with ↑child-responsible attributions and no association with parental self-efficacy
2	General sample of community mothers	Presence of clinically elevated symptoms (yes/no) moderated the association between mothers’ parental attributions and professional help-seeking intentions	Partially supported: No evidence of moderation for child-responsible attributions. For parental self-efficacy, an opposite relationship pattern emerged between groups; however, not clear whether relationship was significant in non-clinically elevated group
3	Subgroup of mothers reporting on a child with clinically elevated mental health symptoms	Omnibus test of whether mothers’ parental attributions was associated with professional help-seeking intentions. If supported, ↑professional help-seeking intentions was associated with ↓child-responsible attributions and ↑parental self-efficacy	Omnibus test was supported Results contrary to hypothesis: ↑professional help-seeking intentions associated with ↑child-responsible attributions and ↓parental self-efficacy
4	Subgroup of mothers reporting on a child with clinically elevated mental health symptoms	Problem type and severity moderated the association between mothers’ parental attributions and professional help-seeking intentions	Not supported

The current findings suggest that mothers’ parental attributions influence their intentions to seek and engage professional help. Comparing results to previous research, however, requires us to consider the influence of child-responsible attributions and parental self-efficacy separately. The finding that child-responsible attributions were significantly influential whereby professional help-seeking intentions appears most likely when mothers attribute child problems to controllable-stable factors provisionally converge with findings from Maniadaki et al.'s [21] study, although differences in constructs measured prevent meaningful comparison of results. However, the current finding is inconsistent with previous research that used vignettes to assess parents’ causal beliefs which found no relationship between variables [22, 23]. Comparisons suggests that the influence of child-responsible attributions on professional help-seeking intentions may be detectable when attributions are based on parents’ own parenting experiences [25]. Importantly, the current study indicated that the association between child-responsible attributions and professional help-seeking intentions was not dependent on the presence of clinically elevated child mental health symptoms suggesting the finding relates to general populations of community mothers.

By contrast, findings related to mothers’ parental self-efficacy suggest a nuanced relationship with professional help-seeking intentions. For instance, parental self-efficacy was not associated with professional help-seeking intentions when examined in a general sample of community mothers, which converges with previous studies drawing from similar populations [21, 24]. However, lower parental self-efficacy was associated with greater professional help-seeking intentions in a sample of mothers reporting on a child with clinically elevated mental health symptoms, albeit at a less stringent level of significance. Nevertheless, the result converges with research examining actual professional help-seeking behaviour [14, 17]. Mothers may be more willing to seek and engage professional help when parenting a child with elevated mental health problems leads to a lower sense of parenting competence which motivates them to seek external professional help [17]. Finally, an anomalous result from our analysis should be noted in that there was evidence of a possible opposite relationship among mothers reporting on a child with symptoms in the normal range whereby higher parental self-efficacy appeared to be associated with greater professional help-seeking intentions. However, replication will be required before interpretations can be made given there was no main effect for parental self-efficacy in the general community sample.

The current results are consistent with help-seeking models proposing that parental attributions for child problems contribute to explaining individual differences in parents’ willingness to seek and engage professional help [7–9, 13]. Findings were consistent even when problem type and severity were considered. However, the direction of influence was in contrary to expectations [13]. The current results suggest that it was a negative rather than positive attributions style that was associated with higher professional help-seeking intentions. Divergent outcomes from expectations may relate to differences in examining actual/past behaviour versus future behavioural intentions [cf. 15]. In line with this, mothers may have more intentions to seek and engage professional help when they hold cognitive stances of blame and helplessness that contribute to an overall low expectancy of being able to manage child problems that leads them to endorse the need to seek professional help [43, 44]. Further research is needed to test this and to disentangle the direction of influence of parental attributions on help-seeking intentions.

More broadly, the results preliminarily support the need to educate community parents about the causes of child problems to improve appropriate professional help-seeking for child mental health [3]. Tully et al. [3] suggest including biopsychosocial explanations for childhood disorders may help to reduce stigma and empower parents to become agents of change. The current results suggest this may also involve targeting the extent to which parents ascribe causality for mental health problems to the child or themselves to help parents make effective decisions about the type of help required to meet child mental health need [45]. Additionally, increasing knowledge about symptoms and helping parents differentiate emerging mental health problems from developmentally normal challenges, alongside educating parents about causal factors as part of improving child mental health literacy, may further encourage professional help-seeking for child mental health [3].

We note there are several limitations in the current study that may present as future directions for research. First, future research should include an examination of fathers’ help-seeking intentions. Previous reviews have indicated that the presence of fathers in the family may moderate help-seeking behaviour [40] and other reviews have highlighted the need to consider fathers in models of treatment engagement due to underrepresentation [46, 47]. Further, research shows that fathers’ attributions have distinct effects on child outcomes unique from the effects of mothers’ attributions [48]. Thus, fathers’ potential unique influence on help-seeking warrants investigation. Notwithstanding this current limitation, there is substantial motivation for examining mothers. Research examining parental roles in families have found that, while fathers are more involved in parenting than ever before, mothers still spend more hours caring for children than do fathers’ and their role tends to expand to crisis managers during times of family distress, including seeking help and making referrals to child health services [13, 49–51].

The second key limitation for future research to address relates to risk of bias. Namely, there was the risk of selection bias from potential individual differences between participating versus nonparticipating mothers [52, 53]. Further, the use of self-report scales may have led to response bias and allowance for mothers to complete the survey at their convenience may have led to diversified patterns of item responses [i.e., administration bias; [54]]. There was also the risk of coverage bias in the absence of response rate information and that most mothers in this study were professionals, well educated, living in metropolitan urban areas, and were from Anglo-European backgrounds [53]. There is a clear need to examine behavioural intentions in a representative sample of parents from diverse backgrounds. Finally, there was potential estimation bias in regression modelling as we did not examine the influence of other parental cognitions that may contribute to motivational processes in service utilisation, namely problem recognition, perceived need, and stigma [3, 55]. Some measures in the model were also not psychometrically evaluated (e.g., mothers’ emotional health; PLOC-SF) which could have led to higher measurement error in the model [56]. It should be noted that while these potential biases are important considerations, the current study followed similar sampling procedures documented in previous research involving general samples of community parents [21, 22]. In relation to survey design, we used brief versions of measures to ensure the survey was short as possible to maximise completion rates in accordance with best-practice [57]. This was important as previous research had not examined parents’ cognitive representations of child problems related to their own personal experiences in a community sample of parents with sufficient sample size to test hypotheses regarding motivational processes [11, 12]. Overall, the current study represents a novel step in improving our understanding of the processes involved in parents’ willingness to seek professional help for their child’s mental health.

In conclusion, the current study examined the influence of mothers’ parental attributions on intentions to seek and engage professional help for the purpose of informing strategies to improve parents’ willingness to engage professional help for child mental health. The results support models proposing that parental attributions explain individual differences in parents’ intentions to seek and engage professional help. Mothers’ intentions to engage professional help appeared to be consistently greater when they attributed their child’s problems to child controllable-stable causal factors and this was not dependent on the presence of clinically elevated child mental health symptoms, problem type, or severity. Lower parental self-efficacy also appeared to have a unique influence in increasing mothers’ intentions to engage professional help, but only in the context of parenting a child with clinically elevated mental health symptoms and not in a general sample of community mothers. The former finding was not dependent on problem type or severity. Taken together, we suggest that educating community parents about the causes of mental health as part of improving child mental health literacy has great potential to improve appropriate treatment engagement with child mental health professional services.

## Electronic supplementary material

Below is the link to the electronic supplementary material.

Supplementary file1 (TIF 189 KB)

Supplementary file2 (DOCX 128 KB)

## Footnotes

1 Test–retest reliability calculation based on n = 19 mothers who participated in the survey at two timepoints with approximately eight weeks between administrations (range = 6.82–12.68 weeks). Details of data collection for calculating test–retest reliability are reported in [37]
