# Supplementary figures and images for: Bridging the gap between child mental health need and professional service utilisation: Examining the influence of mothers’ parental attributions on professional help-seeking intentions
Source: Eur Child Adolesc Psychiatry. 2020 Nov 19;31(2):239–51. doi: 10.1007/s00787-020-01682-6 (PMC8837521; doi:10.1007/s00787-020-01682-6)

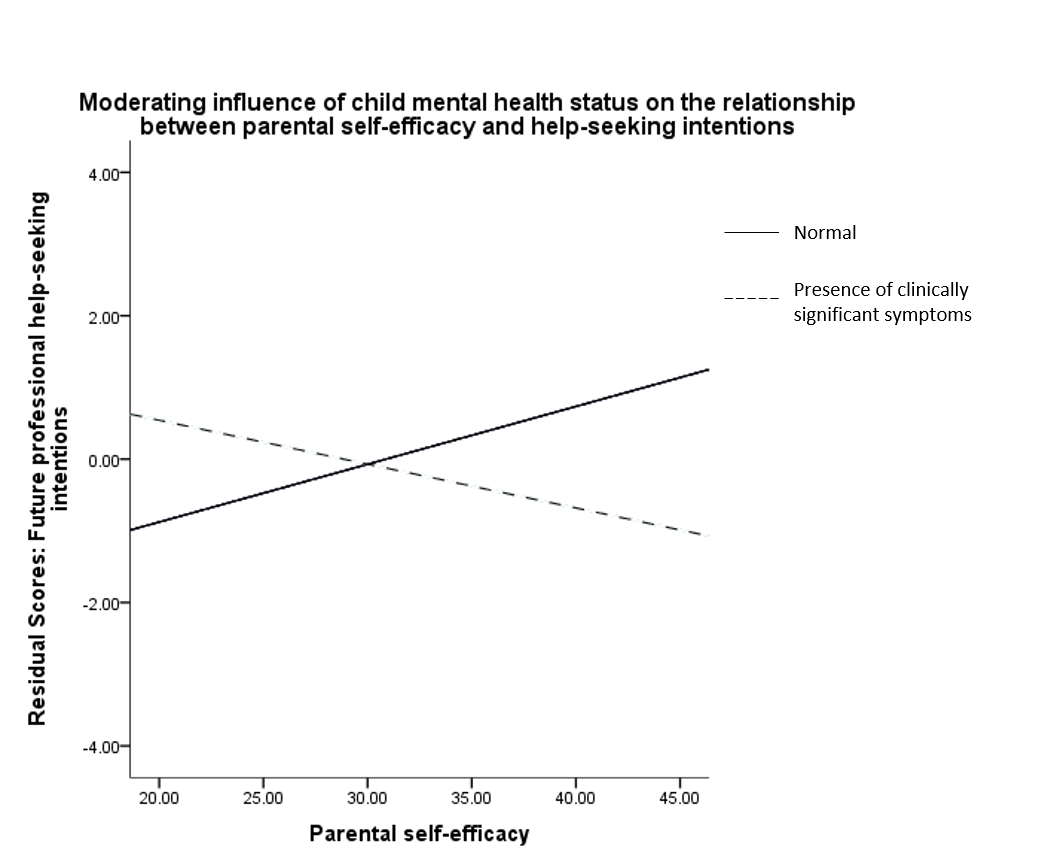

Supplement: Supplementary file 1 — Supplementary file1 (TIF 189 KB) [file 787_2020_1682_MOESM1_ESM.tif]
