# Supplementary material for: Bridging the gap between child mental health need and professional service utilisation: Examining the influence of mothers’ parental attributions on professional help-seeking intentions
Source: Eur Child Adolesc Psychiatry. 2020 Nov 19;31(2):239–51. doi: 10.1007/s00787-020-01682-6 (PMC8837521; doi:10.1007/s00787-020-01682-6)
Supplement: Supplementary file 2 — Supplementary file2 (DOCX 128 KB) [file 787_2020_1682_MOESM2_ESM.docx]

Supplementary Table 1.

*Correlations among variables used to study intentions to engage professional help in the community sample.*

|  | 1 | 2 | 3 | 4 | 5 | 6 | 7 | 8 | 9 | 10 | 11 | 12 | 13 |
| --- | --- | --- | --- | --- | --- | --- | --- | --- | --- | --- | --- | --- | --- |
| 1. Single Parent Status | 1.00 |  |  |  |  |  |  |  |  |  |  |  |  |
| 2. Depression/Anxiety | .12 | 1.00 |  |  |  |  |  |  |  |  |  |  |  |
| 3. Education level | -.03 | -.03 | 1.00 |  |  |  |  |  |  |  |  |  |  |
| 4. Child gender | -.07 | .10 | .14 | 1.00 |  |  |  |  |  |  |  |  |  |
| 5. Child age | -.01 | -.08 | -.02 | -.04 | 1.00 |  |  |  |  |  |  |  |  |
| 6. SDQ Conduct Problems | -.10 | .03 | -.04 | -.12 | -.01 | 1.00 |  |  |  |  |  |  |  |
| 7. SDQ Emotional Symptoms | -.06 | .05 | -.09 | -.03 | .10 | .11 | 1.00 |  |  |  |  |  |  |
| 8. SDQ Hyperactivity | -.01 | .13 | -.17* | -.02 | .07 | .34* | .10 | 1.00 |  |  |  |  |  |
| 9. SDQ Peer Problems | -.04 | -.16* | -.13 | .04 | .07 | .13 | .29* | .22* | 1.00 |  |  |  |  |
| 10. Child-responsible attributions | -.05 | .04 | -.10 | -.06 | .19* | .40* | .14* | .30* | .16* | 1.00 |  |  |  |
| 11. Parental locus of control | .12 | -.11 | -.02 | -.02 | .04 | -.39* | -.18* | -.34* | -.12 | -.43* | 1.00 |  |  |
| 12. Previous professional help-seeking experience | .04 | .12 | -.01 | -.11 | .05 | .03 | .19* | -.05 | .04 | -.08 | .07 | 1.00 |  |
| 13. Future professional help-seeking intentions | .03 | .11 | -.08 | .05 | .06 | -.02 | .19* | .01 | .07 | .10 | -.01 | .69* | 1.00 |

*Note*. * *p* < .05


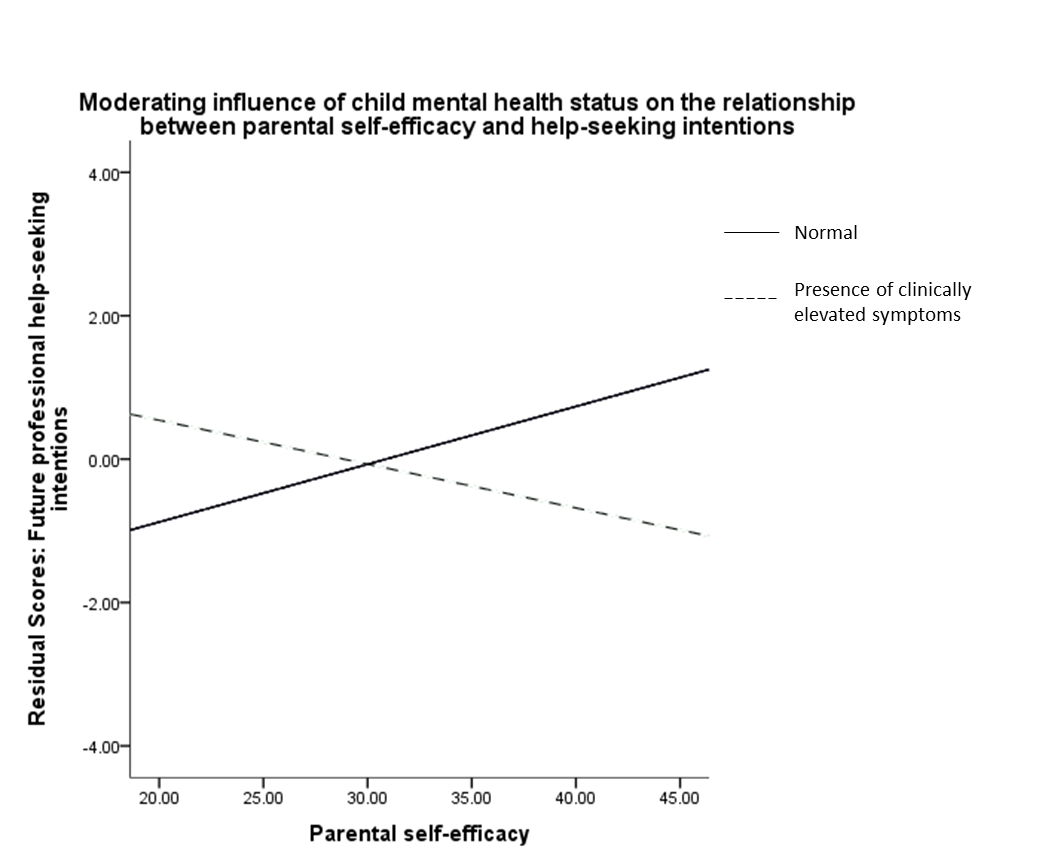


*Figure S1.* Moderating influence of child mental health status on the relationship between parental self-efficacy and future professional help-seeking intentions.

*Note*. Residual scores of future professional help-seeking intentions were calculated from regressing raw scores of future professional help-seeking intentions on previous professional help-seeking experience, single parent status, education level, depression/anxiety, child age, child gender, and child-responsible attributions.
